# Feasibility of an adjunctive INtervention for Debilitating symptom complexes attributed to ticks (FIND): study protocol for a randomised, waitlist-controlled feasibility trial

**DOI:** 10.1136/bmjopen-2025-112627

**Published:** 2026-03-10

**Authors:** Richard A A Kanaan, Richard Macdonell, Michelle Long, Robert J Richardson, Caitlyn Rogers, Mualla Mcmanus, Sabine Braat, Sophie Zaloumis, Cathrine Mihalopoulos, Mary Lou Chatterton, Katherine B Gibney, Georgina Oliver, Sarah J Wilson, Trudie Chalder

**Affiliations:** 1 Department of Psychiatry, University of Melbourne, Heidelberg, Victoria, Australia; 2 Department of Neurology, Melbourne Health, Melbourne, Victoria, Australia; 3 Karl Mcmanus Foundation, Sydney, New South Wales, Australia; 4 Centre for Epidemiology and Biostatistics, University of Melbourne School of Population and Global Health, Carlton, Victoria, Australia; 5 Methods and Implementation Support for Clinical and Health (MISCH) Research Hub, University of Melbourne, Melbourne, Victoria, Australia; 6 School of Public Health and Preventive Medicine, Monash University, Clayton, Victoria, Australia; 7 Department of Infectious Diseases, University of Melbourne at the Peter Doherty Institute for Infection and Immunity, Melbourne, Victoria, Australia; 8 Victorian Infectious Diseases Unit, Royal Melbourne Hospital at the Peter Doherty Institute for Infection and Immunity, Melbourne, Victoria, Australia; 9 Melbourne School of Psychological Sciences, The University of Melbourne, Parkville, Victoria, Australia; 10 Victorian Collaborative Centre for Mental Health and Wellbeing, Carlton, Victoria, Australia; 11 Department of Psychological Medicine, Institute of Psychiatry Psychology and Neuroscience at King’s College London, London, UK

**Keywords:** clinical trials, psychosocial intervention, post-infectious disorders

## Abstract

**Background:**

Debilitating Symptom Complexes Attributed to Ticks (DSCATT) is a new term for an unexplained Australian syndrome—people who suffer from a chronic, multifaceted and debilitating illness, characteristically attributed to tick bites, but in a country without endemic Lyme disease. Despite the profound morbidity of DSCATT, no single causative agent has been identified and there are no recognised treatments for the illness at present. An increasing body of evidence shows psychological therapies such as Acceptance and Commitment Therapy (ACT) can be effective in reducing symptom-related disability and improving quality of life for other unexplained syndromes. Here we present a study protocol to assess the feasibility of an ACT-informed intervention for patients suffering from DSCATT, to be used adjunctively to their pre-existing healthcare. The study aims to assess the acceptability, practicality and demand for the treatment. Additionally, we will examine the effects of therapy on participants’ health and well-being, its safety, potential mediators of response to therapy and its preliminary cost-effectiveness.

**Methods:**

We will assess the feasibility of a 32-week, randomised, waitlist-controlled, parallel convergent mixed-methods pilot trial for DSCATT. Participants will be randomised in a 1:1 ratio to receive either 16 sessions of ACT-informed therapy adjunctive to their pre-existing healthcare, delivered one-to-one with a trial therapist within a 20-week period or be assigned to the waitlist control group where they will continue their treatments as usual. We will collect quantitative and qualitative data to address study aims, with retention rate being the primary feasibility outcome.

**Ethics and dissemination:**

The study has ethical approval from Austin Health Human Research Ethics Committee (HREC). The outcomes will be published in peer-reviewed journals. Data from participants who have given extended consent will be available for other HREC-approved studies.

**Trial registration number:**

ACTRN12623000372684, prospectively registered 13 April 2023, URL: https://www.anzctr.org.au/Trial/Registration/TrialReview.aspx?id=385579&isReview=true; the last participant is expected to complete in November 2026.

**STRENGTHS AND LIMITATIONS OF THIS STUDY**

- First proposed clinical trial for Debilitating Symptom Complexes Attributed to Ticks (DSCATT).
- Mixed methods design to comprehensively explore the feasibility of a proposed intervention for DSCATT.
- Patient-centred psychology intervention to be used alongside pre-existing healthcare (excluding other psychological therapies).
- No agreed case definition for DSCATT exists; inclusion criteria for this trial established by modified Delphi process.
- No agreed outcome measure for DSCATT exists; exploratory DSCATT symptom scale developed for this trial.

## Introduction

 Ticks are parasites that feed on animal and human blood and can cause a range of illnesses to their hosts. Lyme disease is perhaps the most famous tick-borne disease, caused by infection with the bacteria *Borrelia burgdorferi* sensu lato complex, and is endemic in large parts of the northern hemisphere. In Australia, ticks can cause Rickettsial infections, allergic reactions and Mammalian Meat Allergy,^1^ but while many people have developed a ‘Lyme-like’ illness, none has been definitively attributed to Lyme disease acquired in Australia.^2^,^4^ No other infective cause has been identified for these cases following national accredited testing procedures,^4 5^ and while research surveying Australian tick populations has revealed novel bacterial and viral profiles,^6 7^ their impacts on human health are yet to be elucidated.

The presence, or absence, of Lyme disease in Australia has been a highly contentious and polarising issue. After a senate inquiry into Australian Lyme-like illness in 2016,^8^ the Australian Government recognised that there are a cohort of patients suffering from the symptoms of a chronic debilitating illness which many associate with tick bites. They described this patient group as having Debilitating Symptom Complexes Attributed to Ticks (DSCATT), in an attempt to acknowledge this patient group’s multifaceted illness and mitigate the stigma and controversy associated with Lyme terminology, such as Chronic Lyme Disease.

The prevalence and burden of DSCATT is unknown, though the Senate enquiry suggests a large number of people are profoundly affected.^9^ In addition, the pathophysiology of the illness is unclear, and diagnostic biomarkers and symptom criteria are yet to be established. After extensive medical investigations, individuals with DSCATT are usually left without a clear explanation for their illness and often feel that their symptoms and level of impairment are not believed by healthcare providers.^10^ We examined the records of 29 people with DSCATT and found no alternative medical explanation for their extensive and disabling symptoms in the great majority of cases,^11^ so that DSCATT may at present be considered a medically unexplained syndrome (MUS). Many had been diagnosed with various other MUS on their journey, so that its relationship with these is also uncertain.

There are no recognised treatments for DSCATT; however, there has been much research into treatments for other MUS.^12^,^14^ Even without established pathophysiological mechanisms, symptomatic and supportive care may improve symptom-related disability and quality of life. An increasing body of evidence shows that psychological therapies such as Acceptance and Commitment Therapy (ACT) can be effective in this way in MUS,^15^,^18^ as well as in other chronic health conditions such as cancer, chronic pain and stroke.^19 20^ ACT is a ‘transdiagnostic’ intervention which can be applied to individuals struggling with unwanted internal experiences (eg, physical sensations, unpleasant thoughts/feelings), regardless of their health condition or diagnosis.^21^ At its core, ACT aims to improve ‘psychological flexibility’ in the individual and equip them with the practical skills to take effective action to live according to their values, even in the face of pain and suffering.^22^ ACT may be particularly suited for patients with DSCATT by, for example, assisting them to identify meaningful personal values and develop goals matched to those values. Working with people who identified as having DSCATT, we developed a DSCATT-specific treatment informed by ACT, though including other therapeutic elements, and piloted the therapy in seven people who identified as having DSCATT (ACTRN12621001032842; manuscript in submission). Feedback was largely positive and those who completed the programme said they would recommend it to others.^23^

In this study, we propose a feasibility trial, given there are no published clinical trials or treatment reports for DSCATT, and given the controversy that can accompany psychotherapeutic interventions for MUS. As such, there is uncertainty around (a) the demand for, and acceptability of this type of treatment approach for DSCATT; (b) whether therapists will be able to deliver the therapy as per a standardised manual and (c) whether DSCATT patients will engage with the therapy itself. Further, there is uncertainty as to the value of the intervention to participants, and the measures that should be used to assess treatment efficacy. Therefore, we first need to assess if our therapy is practical, acceptable and in-demand for patients with DSCATT before a larger scale, treatment efficacy trial is conducted. Completion of this feasibility trial may also assist in identifying: (a) an appropriate primary outcome measure for future trials; (b) identifying factors which influence response to treatment (including expectations of treatment, and cognitions and behaviours related to illness); (c) changes in brain activity following treatment and (d) the preliminary cost-effectiveness data of the intervention.

We will use quantitative and qualitative data collection methods following a mixed-methods, parallel convergent design to provide a comprehensive and detailed understanding of the feasibility of the therapy for DSCATT. Further, this approach will provide insight into the effects of the therapy on patients’ health and well-being in the context of their DSCATT illness, and its safety profile.

## Study objectives

### Primary aim

Assess the feasibility of a 16-session ACT-informed treatment programme for adult patients with DSCATT. Markers of feasibility include practicality (including eligibility rate and treatment fidelity), acceptability (retention rate and satisfaction with treatment) and demand (including inquiry and participation rates). See table 1 for all feasibility markers used in the trial.

**Table 1:** Feasibility outcomes

Area of focus	Feasibility research question/aims	Outcome measure and data source (quantitative or qualitative)	
		Quantitative	Qualitative
Acceptability	To what extent is the therapy judged as suitable, satisfying or attractive to participants with Debilitating Symptom Complexes Attributed to Ticks (DSCATT)?	Number of participants who do not withdraw prematurely from the programme (retention rate*) Treatment satisfaction questionnaire (satisfaction rate)	Reasons for premature withdrawal (field notes kept by project coordinator and/or trial therapist; semi-structured participant feedback interview) Satisfaction with treatment explored in semi-structured, post-treatment feedback interview
	What is the ability of participants to engage with the programme?	Number of sessions attended by participants (attendance rates)	Explored in semi-structured, post-treatment feedback interview
Demand	To what extent is the therapy likely to be used by patients with DSCATT (ie, how much demand is likely to exist?)	Number of people who express interest in the study (inquiry rate) Number of people who consent to participate in the therapy (participation rate)	Reasons for declining to proceed with the trial, obtained where possible (field notes kept by project coordinator)
Practicality	Is our screening process suitable to identify eligible candidates for randomisation into the trial?	Number of participants who are screened and deemed eligible for the trial (eligibility rate)	–
	Can the intervention be delivered as per a standardised therapist manual?	Therapist adherence to the intervention (treatment fidelity)	–
	Is it feasible to collect health service use via participant self-report and Medicare Benefits Scheme/Pharmaceutical Benefits Scheme (MBS/PBS) data access?	Completion rates of the self-report resource use questionnaire (RUQ) Number of participants who consent to MBS/PBS data access	–

* Primary outcome; measures adapted from Refs.^39^,^41^

### Secondary aims

Examine the effects of the therapy on participants’ health and well-being, and its safety profile (see table 2).

**Table 2:** Clinical, safety and exploratory outcomes

Area	Measure	Timepoint
Clinical		
Quality of life	Assessment of Quality of Life 8-dimension scale (AQoL-8D)^42 43^	Baseline, Week 10, 20 and 32
Functioning	Work and Social Adjustment Scale (WSAS)^44^	Baseline, Week 10, 20 and 32
Depression, anxiety and stress symptoms	Depression, Anxiety and Stress Scale 21 (DASS-21)^45^	Baseline, Week 10, 20 and 32
Severity of illness and perceived improvement	Patient Global Impression scale (PGI)—severity and improvement^46 47^	Baseline (severity subscale only), Week 10, 20 and 32
Physical symptoms	30-item participant self-report Debilitating Symptom Complexes Attributed to Ticks (DSCATT) symptom scale developed by our research group	Baseline, Week 10, 20 and 32
Psychological flexibility	Comprehensive assessment of Acceptance and Commitment Therapy processes (CompACT)^48^	Baseline, Week 20 and 32
Clinician* rated severity of illness and perceived improvement	Transdiagnostic Clinical Global Impression (T-CGI) scale^49^	Baseline (severity subscale only), and treatment endpoint, that is, after completion of the participants 16th treatment session
Safety		
Safety of the intervention	Adverse events, as reported by the participant, and/or as observed by study staff	Assessed at each session by trial therapists; assessed at Week 10 and Week 20 by project coordinator (waitlist controls)
	Negative Effects Questionnaire (NEQ)—20 item^28^	Postintervention (Week 20) for therapy randomised, and treated waitlist controls
Exploratory		
Preliminary cost-effectiveness of the intervention	Healthcare resource use and cost, and lost productivity (absenteeism and presenteeism). Obtained via participant self-report on a tailored resource use questionnaire (RUQ) and supplemented by Medicare Benefits Scheme/Pharmaceutical Benefits Scheme (MBS/PBS) data from Services Australia (where specific consent is provided to access this data).	Baseline, Week 20 and Week 32 (RUQ); MBS/PBS data obtained for 3 months prior to randomisation (to reflect the same reporting time frame as RUQ) up until 8 months post randomisation
Brain activity	Task-based (emotional Stroop) and resting-state fMRI Blood Oxygen Level Dependent (BOLD) signal	Baseline and post-treatment (20 weeks after completion of the intervention)
Mediators of treatment response	Treatment expectancy measured by the Credibility Expectancy Questionnaire (CEQ)^50^	Baseline
	Cognitions and behaviours, as measured by the Cognitive Behavioural Response Questionnaire (short version; CBRQ-S),^51^ and the CompACT	Baseline and Week 20
Changes in clinical status of treated waitlist control participants	AQoL-8D, DASS-21, WSAS, physical symptoms, PGI data	Week 32 of RCT phase (treated waitlist controls’ baseline), and 10 weeks and 20 weeks after commencing the therapy

All measures are participant self-report, unless otherwise specified; baseline=prior to randomisation.

* Rated by the participant’s trial therapist.

RCT, Randomized controlled trial.

### Exploratory aims

Explore potential mediators of treatment response (treatment expectancy, and cognitions and behaviours), cost-effectiveness of the intervention and functional neuroimaging correlates of DSCATT and response to therapy (see table 2). In addition, we will explore changes in clinical outcomes over time in treated waitlist control participants.

### Hypotheses

We hypothesise that the therapy will be practical, acceptable and in-demand for people with DSCATT. Given this is a feasibility trial, any hypothesis testing between the treatment groups will be limited and will therefore be interpreted alongside the magnitude and direction of the treatment effect estimate and two-sided 95% CI (where applicable).

## Methods and analysis

### Trial design

A 32-week, individually randomised, waitlist-controlled, parallel convergent mixed-methods feasibility and pilot trial involving adults (≥18 years) located in Australia with DSCATT. This study will collect quantitative and qualitative data to address its study aims.

Participants will be randomly assigned in a 1:1 ratio to receive 16 sessions of ACT-informed therapy, delivered one-to-one by a trial therapist within a 20-week period and adjunctive to their pre-existing healthcare with follow-up at 32 weeks OR participants will be assigned to the waitlist control group where they will continue their treatment as usual for 32 weeks. After control participants complete the 32-week waitlist, they will be offered access to the 16-session therapy with a trial clinician.

### Participants

#### Eligibility criteria

The trial will recruit adults 18 years of age and older who have access to Telehealth facilities or are able to attend on site at the Austin Hospital (Heidelberg, VIC, Australia). Participants will be assessed by a trial physician to determine if they meet the research case definition for DSCATT. The case definition was developed by our research group via a modified Delphi process involving clinicians, researchers and patient representatives, in combination with analysis of a DSCATT patient and healthy control self-report symptom dataset. Additional eligibility criteria are outlined in table 3.

**Table 3:** Eligibility criteria

Inclusion criteria	Exclusion criteria
Able to consent to the study Adults ≥18 years of age Meet the trial’s case definition for Debilitating Symptom Complexes Attributed to Ticks (DSCATT): *DSCATT requires evidence of a tick bite* which must be: Subject report of an observed tick bite (though symptoms may not develop for several months afterwards) OR serological results from a NATA-accredited laboratory consistent with previous infection (ie, detection of pathogen-specific IgG) with a tick-transmitted pathogen, for example, *Rickettsia*, Q-fever, *Babesia*, *Anaplasma*, *Borrelia* or tick-borne encephalitis virus DSCATT can be acquired in *Australia or overseas* DSCATT *must involve symptoms in three or more systems* (cardiovascular, musculoskeletal, neurological, etc) which *must include fatigue and a cognitive* *symptom* DSCATT symptoms can be mild, but *must be present for at least 6* months, including relapse and remission and *must impact everyday* *activities* DSCATT can develop following one or more diagnosed infections, or without diagnosed infection DSCATT is excluded by other medical conditions which better explain the symptoms, but not by abnormal tests in isolation, nor by other medically unexplained syndromes Other supporting (although not mandatory) criteria: DSCATT can involve pain DSCATT can involve neuropathy (weakness, nerve pain, numbness or tingling) A person with DSCATT may have had a rash (including but not limited to, erythema migrans) as part of their illness Symptoms of DSCATT can vary in type and intensity, within the same person over time. Access to Telehealth facilities/able to attend on site. Possess English sufficient to engage in the treatment programme and answer self-report questionnaires. Able to pause any existing psychological therapies during the treatment intervention.	Current psychotic disorder as per Mini International Neuropsychiatric Interview (MINI) assessment. Diagnosis of a medical condition which better explains the participant’s symptoms (excluding medically unexplained syndromes/functional disorders).

* This case definition was developed in 2022 and updated in 2024 following a modified Delphi process facilitated by our research group involving patient representatives, clinicians and scientists familiar with DSCATT.

NATA, National Association of Testing Authorities (Australia).

#### Study setting

The trial will be conducted remotely via telehealth, or in-person at the Austin Hospital for those who prefer to attend on site. An optional pre–post-treatment neuroimaging component will be conducted at the Melbourne Brain Centre Imaging Unit (MBCIU; University of Melbourne, Parkville, VIC, Australia).

#### Recruitment

Recruitment is anticipated to occur from June 2023 until October 2025. The trial will be advertised via a patient advocacy and tick-borne illness education organisation, the Karl McManus Foundation, who will circulate Human Research Ethics Committee (HREC)-approved recruitment flyers to their members. Clinicians and researchers known to work with DSCATT patients may also distribute these flyers to suitable candidates with a study invitation letter, if desired. In addition, the trial website will be listed on the Australian Government Department of Health’s tick bite associated illness in Australia website. If required, we will engage a clinical trial recruitment agency to obtain suitable candidates.

Study staff will respond to inquiries and address queries where applicable (via telephone/email, as preferred by the person inquiring). Candidates interested in participating in the study will be briefly telephone screened by the project coordinator to ascertain their age, diagnosed medical conditions, current symptoms and treatments. They will be sent the participant information and consent form (PICF) via email/post (as they prefer) and be asked to review in their own time. A suitable time to follow-up with the candidate will be arranged to address any further queries, and to book in their medical screening appointment if they wish to proceed with the trial.

#### Consent

Written, informed consent will be required for study participation, and prior to completing the medical screening appointment to assess eligibility. PICFs will be issued to study candidates by the project coordinator via email/post, as preferred (see online supplemental material ‘PICF Master FIND trial V.3.3’). After reviewing the PICF, candidates may have as much time as desired while recruitment for the trial is open to consider participating. Informed consent procedures may be completed by the project coordinator or the trial physician. Participants will have the option to consent via one of three ways: (1) Face-to-face with study staff, (2) E-consent via Research Electronic Data Capture (REDCap),^24 25^ (3) Informed consent completed via telehealth/telephone, and a signed hard copy of the consent form returned via post or scanned and emailed to study staff. All consent forms will be co-signed by study staff who completed informed consent with the participant, and a copy of the dually signed consent form will be sent to the participant.

Participants may be asked to re-consent to the study if terms of consent change. For example, if study procedures are amended during the trial which affects how participant data is used, participants will be contacted to discuss this change and be asked to sign an updated PICF, if willing.

##### Optional study components requiring consent

Participants able to attend MBCIU in Parkville, VIC may provide additional consent to partake in the neuroimaging component of the study. The study PICF will contain a separate consent section specific to this component. A PICF supplied by Services Australia will be required for participants who wish to provide their Medicare Benefits Scheme (MBS) and Pharmaceutical Benefits Scheme (PBS) data to be used as part of the health economic evaluation of the study (see the Study procedures, Explatory procedures section). In addition, participants may provide extended consent for their study data to be shared to future projects that are ‘(1) an extension of, or closely related to, the original project; or (2) in the same general area of research (eg, genealogical, ethnographical, epidemiological or chronic illness research)’.^26^ These projects must have received approval from a registered HREC committee. Data will be shared in a non-identifiable format where possible.

### Intervention

#### Therapy arm

Therapy will consist of 16 sessions delivered by a psychologist via Telehealth or face-to-face at the Austin Hospital. The first session will be 90 min, whereas follow-up sessions will be 60 min. Sessions will be delivered weekly as much as possible; however, participants will have 20 weeks to complete all 16 sessions to accommodate for cancellations.

The therapy is a hybrid, based on principles of ACT, but including several elements from Cognitive Behavioural Therapy. Delivery is guided by a purpose-written therapist manual and corresponding participant workbook. Development of the therapy, including patient involvement and integration of their feedback can be read in detail here (Dharan *et al*, in submission). Manualised modules include (1) education on concepts of health and illness and the neurobiology of symptom experience in DSCATT, (2) core principles of ACT, (3) evaluating the daily routines, (4) increasing levels of activity, (5) identifying and connecting with values, (6) setting goals, (7) strategies for managing uncertainty of illness. Additional modules on self-compassion, improving sleep, addressing difficulties with thinking and memory, and navigating social relationships will be administered according to participant need. See online supplemental table S1 for an outline of content covered in each session.

Trial clinicians will have the capacity to flexibly adapt individual sessions to suit the presenting needs of participants. The participant will decide which is the most pressing issue/problem for them. This will guide the order of delivery of modules between session 6 and 10, contingent on where the participant is currently at in their therapy trajectory.

Participants will be provided with homework tasks each week via email, to be completed between sessions to practise introduced skills and concepts. They will be encouraged to set aside up to 1 hour per week to complete these tasks. Homework worksheets provided to the participants are included in the participant workbook. A reminder email or SMS, based on the participants’ communication preferences, will be delivered during the week to follow-up on homework tasks. A review of homework tasks will be performed each session, including whether the participant completed the set homework and discussion of progress and any challenges experienced.

Treatment adherence (visits attended) will be recorded by the trial therapists. Therapist notes detailing modules addressed in session, homework completed and risk assessment, as per standard clinical practice, will be recorded.

Following completion of the Week 32 questionnaires, trial therapists will contact their participants via telephone to discuss ongoing progress, and answer queries or concerns the participant may have following the cessation of the intervention.

##### Trial therapist eligibility

Sessions will be delivered by Australian Health Practitioner Regulation Agency-registered psychologists (clinical, health or neuropsychologists) who have completed additional training in ACT.

##### Treatment fidelity

All sessions will be audio-recorded, and a proportion (10%) will be assessed for treatment fidelity (ie, therapists’ adherence to the treatment manual) using a standardised schedule developed by coinvestigator TC.

### Waitlist control arm

Individuals who are assigned to the waitlist control group will be encouraged to continue their treatments as usual. They will be sent study questionnaires at Baseline, and 10, 20 and 32 weeks postrandomisation. In addition, the project coordinator will telephone participants at Week 10 and Week 20 to review the occurrence of adverse events (AEs) (to serve as a comparator to the therapy arm). After completion of the Week 32 questionnaires, participants will be offered access to the 16-session therapy, if desired. If they choose to proceed, they will be sent additional study questionnaires to complete 10 weeks and 20 weeks after commencing their first therapy session.

### Standard care and addition to standard care

While there is no defined standard care for DSCATT at present, the therapy and study procedures will be completed alongside participants’ treatment as usual. This includes medication, vitamins, herbal remedies and complementary therapies they may be using. However, participants will not be permitted to employ other psychological therapies while they are completing the 16-session trial therapy. They may use other psychological therapies after they complete the intervention, if desired, or if they are randomised into the waitlist control arm. Candidates who are screened for the study and already employing psychological therapies as part of their standard care will be encouraged to discuss the potential of pausing these therapies with their current healthcare provider(s) prior to randomisation into the trial.

Participants’ concomitant treatment throughout the trial will be determined via a self-report resource use questionnaire (RUQ) provided at baseline, Week 20 and Week 32. This will be supplemented via MBS and PBS data retrieval where participant consent is provided.

### Study procedures

**Figure 1:**
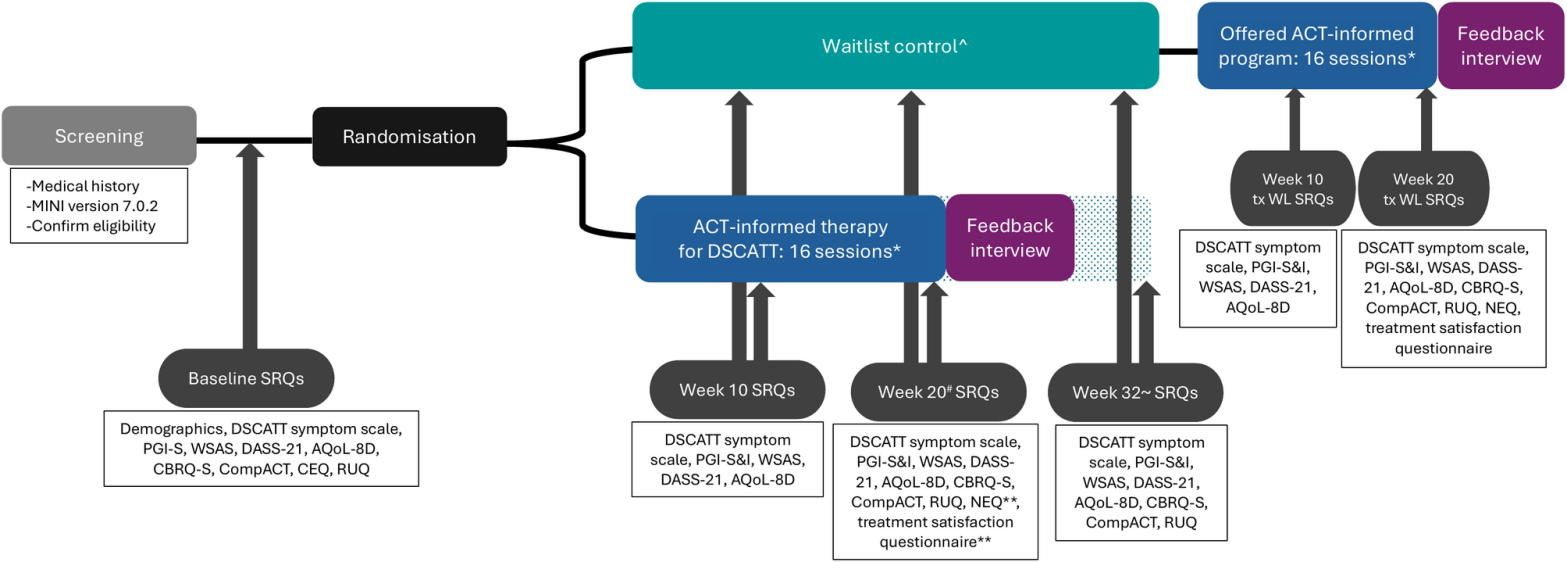
Study procedures following informed consent. ^∧^Project coordinator contacts participant at Week 10 and Week 20 to review for AEs; *AEs reviewed by trial therapist in each treatment session, T-CGI completed by trial therapist pretreatment and post-treatment programme; **treatment programme participants only. Note: self-report questionnaires (during the RCT phase) will be administered at baseline (prior to randomisation), 10 weeks, 20 weeks and 32 weeks postrandomisation (irrespective of what session number the therapy randomised participants are up to in the treatment programme). ^#^Primary endpoint; ^~^follow up timepoint. Neuroimaging at the Melbourne Brain Centre Imaging Unit, Parkville, optional for participants (treatment group and treated waitlist group) to complete prior to commencing the treatment programme and repeated 20 weeks later/after their 16th therapy session—not shown on figure. ACT, Acceptance and Commitment Therapy; AE, adverse event; AQoL-8D, Assessment of Quality of Life-8 dimension; CBRQ-S, Cognitive Behavioural Response Questionnaire (short version); CEQ, Credibility/Expectancy Questionnaire; CompACT, Comprehensive assessment of Acceptance and Commitment Therapy processes; DASS-21, Depression Anxiety Stress Scale, 21 item; DSCATT, Debilitating Symptoms Complexes Attributed to Ticks; MINI, Mini International Neuropsychiatric Interview; NEQ, Negative Effects Questionnaire; PGI-S/I, Patient Global Impression scale (severity and improvement); RUQ, resource use questionnaire; SRQs, self-report questionnaires; T-CGI, transdiagnostic Clinical Global Impression scale; tx WL, treated waitlist; WSAS, Work and Social Adjustment Scale.

See figure 1 for study schema which outlines procedures following informed consent.

#### Screening

Following written, informed consent, participants will be assessed by a physician who will review their medical history, confirm the presence of DSCATT and ensure the participant meets the remaining eligibility criteria (see table 3). Participants’ medical records held by other healthcare providers will be requested as necessary (as specified on the PICF). In addition, the trial physician may order investigations to confirm eligibility (for example, to rule out a medical condition which may better explain their symptoms) on an as-needed basis, with results shared with their treating team. The MINI International Neuropsychiatric Interview (V.7.0.2)^27^ will be administered at this visit. If eligible and willing to proceed, participants will be randomised into the trial by the project coordinator (see the Randomisation section).

#### Study questionnaires

Participants will be sent a suite of questionnaires via REDCap at baseline (prior to randomisation into the therapy/waitlist control arm); and 10 weeks, 20 weeks (primary endpoint) and 32 weeks (follow-up) after randomisation (and irrespective of which session number the participant has completed in the treatment programme). These questionnaires will assess quality of life, general functioning, disability and impairment, physical symptoms, views about illness and healthcare utilisation (refer to the Outcomes section for further details). All participants who partake in the treatment programme (randomised participants and waitlist controls) will be provided with a treatment satisfaction questionnaire and the 20-item Negative Effects Questionnaire (NEQ)^28^ 20 weeks after commencing the therapy to ascertain its acceptability and safety.

#### Feedback interview

All participants who partake in the treatment programme (randomised participants and treated waitlist controls) will be invited to attend a feedback interview with the project coordinator after they have completed their final session (note, participants who are withdrawn prematurely from the trial may also attend this interview if desired). The project coordinator will follow a semi-structured interview guide (see online supplemental material ‘feedback interview guide’) to explore the participant’s views and experiences of the treatment, including perceived value of the therapy. The interview will be audio recorded, transcribed verbatim (names omitted) and then analysed following qualitative analysis methods.

#### Safety monitoring

Our definition, assessment and recording of AEs and harms of treatment will be informed by Klatte *et al*^29 30^ and the National Health and Medical Research Council (NHMRC) safety monitoring and reporting guidelines.^31^ AEs may include worsening of pre-existing symptoms, recurrence of previous symptoms not present/disclosed at randomisation, diagnosis of a new medical condition and/or emergence of new symptoms or injuries.

The occurrence of AEs in participants randomised to receive the therapy will be reviewed by the trial therapists during each treatment session and documented in REDCap as necessary. AEs will include those reported by the participant and those observed by the trial therapist. The severity and causality of events to the therapy, and other study procedures, will be rated by both the participant and the medical monitors. The medical monitors will be unblinded to group allocation so details of the participants progress in therapy can be discussed (as relayed by the participant’s trial therapist) and ascertained if related to the AE. Waitlist control participants will be telephoned at Week 10 and Week 20 by the project coordinator to determine the occurrence of AEs. All AEs will be followed up until stable/resolved. Verbal consent will be required from the participant to be followed up if AEs persist at Week 32.

All safety reporting in the study will follow the Sponsor’s (Austin Health) current safety reporting guidelines.

#### Exploratory procedures

##### Health economic evaluation

Participants may consent to provide access to data on use of healthcare services through MBS and PBS. These data provide detailed information on visits to healthcare providers, diagnostic tests and prescription medications including the amount paid out of pocket by participants and the amount paid by the Commonwealth government. Data will be extracted for the period 3 months prior to randomisation up until 8 months postrandomisation. The MBS/PBS data will be used to supplement self-report health service use obtained in a study-specific RUQ. The RUQ is adapted from previous Australian trials of mental health interventions and assesses the type, frequency and duration of health service use across diagnostic testing, medication use, outpatient, emergency and inpatient care. Questions also gather data on lost paid and unpaid work as well as an estimate of productivity while at work with symptoms (presenteeism).

##### Neuroimaging

Participants who provide optional consent to participate in the neuroimaging component of the study will be required to attend the MBCIU at The University of Melbourne (Parkville) for MRI scanning on two separate occasions. Treatment randomised, and treated waitlist control participants will complete their first scan (baseline) prior to their first treatment session and their second scan after their 16th session, or at Week 20. Prior to each visit, all participants will undergo an MRI safety screen by completing a safety questionnaire and review with radiographers at the MBCIU. By consenting to participate in the neuroimaging arm, participants also give consent for the research team to obtain relevant medical records and to share them with the MBCIU, if required for the purposes of the MRI safety screen. The entire MRI scan will take approximately 60 min, during which functional (fMRI) and structural images of their brain will be collected. While in the scanner, the Emotional Stroop Task will be administered. Participants will be presented with a series of words in different colours, appearing one at a time in quick succession on a screen. Participants will be required to identify the colour of each word as quickly and accurately as possible. Each word will be either a control word or a word related to DSCATT. Accuracy and reaction time will be collected on each trial. At the end of the MRI session, participants will be asked to rate each word on a number of dimensions (eg, emotional arousal, pleasantness/valence, relation to their physical or mental state) in a questionnaire facilitated by REDCap. Questionnaire data will aid the interpretation of task performance and neuroimaging findings.

The Emotional Stroop Task was chosen to investigate the neurobiological mechanisms underlying attentional bias toward information that has emotional relevance to the participant.^32^ Behaviourally, this bias is inferred when participants take longer (increased reaction time) to identify the ink colour of emotionally salient words compared with neutral (control) words. This effect, known as the ‘interference effect’, indicates that emotionally salient information can capture attention and divert cognitive resources away from the task at hand.^33^

We will use the Emotional Stroop task in the scanner to elicit brain activation in response to words describing participant’s symptoms or affective experience of DSCATT, then to compare this data between baseline and postintervention timepoints. We predict that our intervention will help participants shift attention away from symptoms and difficult illness-related thoughts/emotions to other aspects of their awareness. We therefore anticipate that the Emotional Stroop’s ‘interference effect’ will decrease after treatment, accompanied by changes in brain activation between timepoints.

### Outcomes

#### Feasibility outcomes

Table 1 outlines the outcome measures which will be used to assess the feasibility of the therapy for patients with DSCATT, of which retention rate will be the primary feasibility outcome.

#### Clinical, safety and exploratory outcomes

Table 2 outlines all clinical, safety and exploratory outcomes and timepoints where they will be assessed.

### Sample size

#### Sample size estimation

A total of 120 randomised participants is planned to provide adequate precision around the assumption of the primary feasibility outcome and to potentially inform the assumption of the pooled SD needed to work out the sample size of a future trial that is aimed to demonstrate the effect of the therapy. Assuming 120 participants are randomised, if study completion (ie, retention) is observed for 60% of these participants (72/120), then the two-sided 95% CI of the true underlying retention rate is (51%, 69%). The exact (Clopper-Pearson) method was used to obtain the 95% CI. For continuous (normally distributed) outcomes, a sample size of 70 in total (35 per treatment group) is considered sufficient to estimate the pooled SD with reasonable precision.^34^ Regarding the feedback interviews, sampling will continue until theoretical saturation is reached. A review of 100 studies analysing interview data demonstrated that 25 interviews were sufficient to achieve theoretical saturation.^35^ Thus, we anticipate that 25 participants who complete the therapy will be interviewed, and their transcripts analysed.

#### Power calculations

The trial is a pilot and feasibility study and is not designed to detect a treatment effect. As a result, the sample size justification is precision-based and no power calculation was performed.^34^

### Randomisation

A permuted block randomisation technique, stratified by sex at birth (female/male) will be used and accessed only by the project coordinator, who is unblinded during the trial.

#### Allocation concealment mechanism

The randomisation schedule will be computer-generated by a third-party statistician at the Methods and Implementation Support for Clinical and Health (University of Melbourne), not involved in the trial, including its design nor planned analyses. The schedule will be uploaded into the REDCap randomisation module^36^ by the project coordinator. The randomisation schedule and group assignment instruments will be hidden from the trial statisticians in the study’s REDCap project.

#### Implementation

After satisfaction of inclusion criteria and completion of baseline questionnaires, participants will be randomised at a 1:1 ratio to the treatment intervention or waitlist-control arm, adjunctive to their treatment as usual. Randomisation will be completed by the project coordinator using the REDCap randomisation module.

The project coordinator will inform the participant of their group allocation following randomisation and determine their availability to commence sessions if allocated to the therapy arm. The participant will be assigned to a therapist based on matching availability. Participants randomised into the waitlist control group will be reminded of the trial timeline and opportunity to access therapy after 32 weeks.

#### Blinding

Unblinded parties include participants, trial therapists, medical monitors and the project coordinator. Trial therapists will be blinded, however, to participant responses on follow-up self-report questionnaires. They will not have access to Week 10, Week 20 and Week 32 visits in the REDCap participant self-report questionnaire project.

Statisticians will be blinded to participant group assignment while the trial is active. They will become unblinded after database lock.

### Statistical methods

A detailed statistical analysis plan describing the analysis of the feasibility and clinical outcomes will be prepared prior by the trial statisticians to unblinding the database for analysis. All outcomes will be summarised. Descriptive statistics will consist of numbers and percentages for categorical variables. Mean and SD or median and interquartile range (25th–75th percentiles) will be reported for continuous variables. The frequency and percentage of participants with missing data will be provided for all outcomes. Following a mixed-methods convergent parallel design, quantitative data and qualitative data will be analysed separately and results compared with identify how they confirm, contradict or expand on each other.^37^

#### Feasibility outcomes

For binary feasibility markers in table 1, the exact (Clopper-Pearson) method will be used to obtain the two-sided 95% CIs.

#### Clinical outcomes

As the trial is a feasibility and pilot study, reporting of clinical outcomes will be mostly descriptive. Any hypothesis testing between the treatment groups will be limited and will therefore be interpreted alongside the magnitude and direction of the treatment effect estimate and two-sided 95% CI. No adjustment for multiple testing will be implemented due to the non-confirmative nature of the trial. Subgroups (eg, female/male, prior therapy, mode of delivery) will be explored using descriptive statistics only.

Suitability of a clinical outcome measure for a future trial will be gauged via the quantitative analysis outlined earlier and the qualitative analysis of the semi-structured feedback interviews aimed to explore the therapies benefit and potential mechanisms of action. Feedback interview transcripts will be analysed following inductive thematic analysis, as the authors have previously employed.^38^ Assumptions about the pooled SD of the proposed clinical outcome will be derived descriptively, including a two-sided 95% CI for the pooled SD

#### Safety outcomes

AEs will be summarised descriptively by treatment group, including the number of AEs and the number of participants with at least one AE. Summary statistics will be presented for the full measure and for each of the factors of the NEQ.

#### Exploratory outcomes

Among the participants who consent to the neuroimaging component, task-based fMRI run, whole-brain analyses will be employed to determine task-relevant regions of Blood Oxygen Level Dependent signal change using statistical parametric maps computed in SPM12 or other neuroimaging software. Region-of-interest analyses may be additionally employed to interrogate the data. Resting-state fMRI data will be assessed using connectivity analyses. Task-based and resting-state fMRI data will be compared between the pretreatment (baseline) and post-treatment timepoints.

A preliminary evaluation of the likely cost-effectiveness of the therapy will be carried out from partial societal and health sector perspectives drawing on the Assessment of Quality of Life 8-dimension scale (AQoL-8D) utility data, additional outcome measures (ie, Depression Anxiety Stress Scale, 21 item (DASS-21)) and information on health service use and lost productivity collected through a self-report RUQ. The within trial economic evaluation will estimate the cost to deliver the therapy through microcosting. The additional resource use collected through the RUQ will be valued using standard Australian unit costs, and lost productivity will be valued using an average wage rate plus overhead costs. The economic evaluation will first measure and value any change to the use of healthcare resources over the period of the study between the ACT and wait-list groups. Any additional costs associated with the therapy will be compared with the outcomes achieved. Quality-adjusted life years will be derived from the AQoL-8D that will form a cost-utility analysis and the other primary and secondary outcome measures will be used for the cost-effectiveness analysis. Standard economic evaluation techniques will be used including calculation of incremental cost-effectiveness ratios (ICERs) and using non-parametric bootstrapping to determine CIs for ICERs. Sensitivity analyses will be used to determine the impact of varying important study parameters (such as unit cost price variation).

#### Treated waitlist outcomes

In addition to the above-described analysis in all participants using data collected up to and including Week 32, an observational analysis is planned on all data of the waitlist controls who elect to do the therapy. To explore changes over time in treated waitlist controls from baseline up to and including Week 20 post first therapy session, descriptive statistics will be provided at each study visit. For clinical outcomes, these summaries may be complemented with an analysis using a mixed linear regression model that accounts for the longitudinal nature of the data. The feasibility of this statistical analysis will be informed by the sample size among those waitlisted who elect to do therapy. This model will provide estimates, two-sided 95% CIs and p-values of within-group changes in clinical outcomes while waiting and subsequent within-group changes in clinical outcomes following therapy after waiting. Any hypothesis testing will be limited and will therefore be interpreted alongside the magnitude and direction of the treatment effect estimate and two-sided 95% CI. No adjustment for multiple testing will be implemented due to the exploratory nature.

### Data collection and management

Study data will be collected and managed using REDCap electronic data capture tools hosted at The University of Melbourne. This includes participant self-report questionnaires, therapy attendance details, the Transdiagnostic Clinical Global Impression scale and AEs. All data stored in REDCap will be in a re-identifiable format.

The trial therapists will audio record each session with their participants so the therapists’ adherence to the treatment manual and competency can be independently assessed. In addition, treatment feedback interviews will be audio recorded and transcribed using Zoom software and cross-checked by study staff to ensure accuracy of the transcript. All audio recordings and transcripts will be stored on a Records Managed Sharepoint hosted by The University of Melbourne. Hard copy data, including consent forms, clinician notes and the MINI, will be stored in individual participant files in a locked filing cabinet in the Department of Psychiatry, Austin Hospital, VIC, Australia.

All data will be kept for a minimum of 7 years postpublication of the primary paper. After this time, destruction of hard copy data, participant medical records, consent forms and investigator site files will be advised by the Sponsor, Austin Health, and actioned by the Principal Investigator as required. However, non-identifiable datasets used for statistical analysis will be kept in perpetuity to facilitate data sharing and open-access policies as encouraged by the NHMRC (excluding participants’ data where consent for data sharing was not provided).

### Patient and public involvement

Patients and patient representatives were involved in the codesign of the intervention, in the modified Delphi process that produced the case definition, and in the conduct of the trial by representation on the steering group.

## Ethics and dissemination

### Research ethics approval

The study will request ethics approval from Austin Health HREC. Additional amendments to the protocol, PICF, advertising material, etc, will be submitted to the committee for review prior to implementing desired changes, except in the event of urgent safety measures where immediate actions to reduce risk will be implemented.

### Dissemination policy

Publications and supplementary materials will be uploaded into the University of Melbourne Minerva Access repository and Austin Health Research Online repository (author accepted manuscripts where necessary). All publications will follow the International Committee of Medical Journal Editors authorship eligibility guidelines.

A lay, HREC-approved summary of the primary study results will be communicated to participants directly, if desired. A copy of this summary will also be uploaded onto the study’s clinical trial registry listing (Australian New Zealand Clinical Trials Registry; ANZCTR) and provided to the Australian Government Department of Health’s Tick bite diseases and symptoms attributed to tick bites website.

Participants may provide extended consent for their data to be shared to related projects approved by a registered HREC. Data will be provided in a non-identifiable format. A metadata listing will be created in The University of Melbourne’s Melbourne Academic Centre for Health figshare repository outlining the type of data available and requirements to follow before it can be shared (eg, receipt of HREC approval letter to conduct the project).

## Supplementary material

10.1136/bmjopen-2025-112627 online supplemental file 1

10.1136/bmjopen-2025-112627 online supplemental table 1

10.1136/bmjopen-2025-112627 online supplemental file 2

## Data Availability

Data are available upon reasonable request.
