# Supplementary material for: Feasibility of an adjunctive INtervention for Debilitating symptom complexes attributed to ticks (FIND): study protocol for a randomised, waitlist-controlled feasibility trial
Source: BMJ Open. 2026 Mar 10;16(3):e112627. doi: 10.1136/bmjopen-2025-112627 (PMC12983826; doi:10.1136/bmjopen-2025-112627)
Supplement: online supplemental file 1 [file bmjopen-16-3-s001.docx]

**Participant Information Sheet/Consent Form**

**Interventional Study** - *Adult providing own consent*

| **Title** | Feasibility of an adjunctive intervention for Debilitating Symptom Complexes Attributed to Ticks (DSCATT) – a randomised, waitlist-controlled pilot and feasibility trial |
| --- | --- |
| **Short Title** | FIND trial |
| **Protocol Number (Version)** | 1 (3.1) |
| **Project Sponsor** | Austin Health |
| **Principal Investigator** | Professor Richard Kanaan |
| **Location** | Austin Health |

**Part 1 What does my participation involve?**

**1 Introduction**

You are invited to take part in a research project. This is because you have an illness suspected to be caused by tick bites, also known as ‘*Debilitating Symptom Complexes Attributed to Ticks’* (DSCATT). The research project is testing the feasibility of a potential treatment for improving quality of life and day-to-day functioning in people with DSCATT. The new treatment is a psychology-based intervention which has been tailored to suit people who have this illness and complement their existing healthcare.

This Participant Information Sheet and Consent Form tells you about the research project. It explains the tests and treatments involved. Knowing what is involved will help you decide if you want to take part in the research.

Please read this information carefully. Ask questions about anything that you don’t understand or want to know more about. Before deciding whether or not to take part, you might want to talk about it with a relative, friend or your local doctor.

Participation in this research project is voluntary. If you don’t wish to take part, you don’t have to.

If you decide you want to take part in the research project, you will be asked to sign the consent section of this document. By signing it you are telling us that you:

• Understand what you have read

• Consent to take part in the research project

• Consent to have the tests and treatments that are described in this document

• Consent to the use of your personal and health information as described in this document

You will be given a dually signed copy of this Participant Information and Consent Form to keep.

**2 What is the purpose of this research?**

Though DSCATT usually starts after a tick bite, we don’t fully understand why. Many people with DSCATT have completed extensive investigations under different doctors, although are unsure what is causing their symptoms. Though many people who suffer with DSCATT will have tried a range of different treatments, there have been no clinical trials to assess whether they have worked or not. That means we don’t know how to treat it yet. However, research studies have shown that psychology-based interventions can help improve quality of life in people with chronic illnesses, including those with symptoms similar to DSCATT. In this study we want to explore whether this kind of treatment is practical to offer to people with DSCATT (whether it is feasible), and if it will help them manage their symptoms in a way that improves daily functioning and quality of life.

The treatment program that the researchers have developed aims to equip you with practical strategies designed to help you manage barriers and setbacks that you may be encountering as a result of having DSCATT. By the end of treatment program, it is possible that your symptoms will have less impact over your life. While there is no guarantee that symptoms will disappear, research on this kind of treatment has shown many people with various medical conditions will experience improvements in symptom management and severity. The treatment program we are proposing is experimental which means that it is not an approved treatment for DSCATT. Therefore, it needs be tested to see if it is an effective treatment option.

This research study has been funded by The National Health and Medical Research Council (NHMRC) and involves researchers from The University of Melbourne, Austin Health, Monash University and King’s College London.

**3 What does participation in this research involve?**

You will be participating in a randomised, wait-list controlled pilot study. This means that you will be randomly allocated into one of two groups: 1) the treatment program group or 2) the wait-list control group. You won’t be able to choose which group you are in, neither will the researchers. This is decided at random by a computer program to reduce bias and so there are even numbers of participants in each group.

If you are enrolled into the treatment program group, you will start the therapy with a trial clinician as soon as you can. If you are enrolled into the wait-list control group, you will continue with whatever treatment you usually have for 32-weeks (about 7 months). After this wait-list period is over, you can try the treatment program if you wish. The reason why there is a wait-list control group is so that the researchers can compare the group of people who go through the treatment program against a group of people with DSCATT who don’t do the program. This will allow the researchers to see if there are any differences between the groups over time – for example, if symptoms improve more so in one group than the other.

*Screening appointment*

If you would like to take part in this project, you will be asked to read through this information document carefully and then provide written consent before any of the study assessments or treatments are provided. After signing the consent form, you will meet with a medical doctor (a physician) who will review your medical history and mental health to assess if you are eligible to take part in the trial. With your permission, they may request to see some of your medical records held by other healthcare providers to help detail your medical history and avoid you having to repeat things unnecessarily. The appointment with the physician can take place face-to-face at the Austin Hospital, Heidelberg, VIC, or via Telehealth as you prefer. The appointment is likely to take 60-90 minutes.

*Study questionnaires*

If you are eligible for the trial, you will be asked to complete a series of questionnaires which will ask about your demographic information (including your age, postcode, and employment status), your quality of life, physical symptoms, depression and anxiety, healthcare services you use, and views about your illness. These questionnaires will be emailed to you to complete online before you are randomised into the trial (baseline), and 10-weeks, 20-weeks and 32-weeks later. They are expected to take between 30-40 minutes to complete each time. If you don’t have access to email, we can arrange for the questionnaires to be posted to you with a pre-paid reply envelope so you can post them back to the researchers.

*Treatment program*

After you complete your baseline study questionnaires, you will then be enrolled into the treatment program or the wait-list control group. If you are enrolled into the treatment program, you will meet with a trial clinician (a psychologist) one-on-one via Telehealth or face-to-face at the Austin Hospital in Heidelberg, VIC, as you prefer. The program consists of 16 sessions which last approximately 60 minutes each (the first session may take up to 90 minutes) and are typically completed once per week. If you would like more time between sessions, please discuss with your trial clinician. Please note, the maximum amount of time you will have to complete all 16 sessions is 20 weeks. The sessions will be tailored to suit your needs, however, some key things which may be explored is the relationship between thoughts, emotions and behaviours, and how they may contribute to the physical symptoms and chronic nature of DSCATT; developing strategies to help manage difficult symptoms (for example pain, fatigue) as well as emotions (fear, stress, frustration, uncertainty); and identifying things that are important to you (your values) and how you can work towards achieving them. You will be given a participant manual to guide you through the program. You may have some simple activities (e.g., reading material, worksheets) to complete at home in between appointments. These take-home activities allow you to practice the skills you will learn in the weekly appointments, facilitating your progress through the treatment program. The time commitment for these activities can vary from week to week, but on average, we ask that you reserve 1 hour of your week to complete them. We recommend that you complete the activities before your next session to get the most benefit from our program. We will send you an email or SMS (as you prefer) in between sessions as a reminder. The sessions with the trial clinician, and online study questionnaires, will be in addition to your regular treatments – you shouldn’t have to change any of your usual care while you take part in the study.

Please note, your appointments with the trial clinician may be audio recorded. This is for training and supervision purposes of the trial clinician. Your clinician will remind you at the start of each session before they begin recording to ensure you are comfortable with this.

At the end of the program, you will be invited to complete an interview with a research assistant so you can provide feedback about the treatment (for example, what you liked, and/or what you didn’t like). The interview may take up to 1 hour to complete, and it will be recorded and then transcribed word-for-word so it can be analysed in detail by the researchers. The transcripts will be edited so that they do not contain identifying details (for example, people’s names will be removed).

Lastly, approximately 3-months after you complete the treatment program, your trial clinician will contact you via telephone for a brief check in to review your progress and address any queries you may have.

*Wait-list control group*

If you are enrolled into the wait-list control group, you will continue your current treatments and other routines as usual. You will be sent online study questionnaires at baseline, 10 weeks, 20 weeks and 32 weeks later. A research assistant will also contact you via telephone at 10 and 20 weeks to review your health and wellbeing. After you complete your Week-32 questionnaires, you can try the 16-session treatment program if you would like to. If so, we ask that you complete some additional questionnaires 10-weeks and 20-weeks after you start the program (similar to those that you completed during the waitlist period).

**Table 1.** *What does participation in this study involve?*

| **Timepoint in study** | **Details** | **Duration** |
| --- | --- | --- |
| **Screening appointment** | With a medical physician face-to-face or via Telehealth | 60-90 minutes |
| **Baseline questionnaires** | Emailed to you to complete online* | 30-40 mins |
| **Randomised into study**  **(group a or b)** | **a) Treatment program group:** 16-sessions completed one-on-one with a trial clinician via Telehealth or face-to-face at the Austin Hospital | Approximately one session per week; 90 mins for the first session, 60 mins thereafter |
|  | **b) Wait-list control group:** Continue your usual treatments and routines | N/A |
| **Week-10** | Questionnaires emailed to all participants to complete online* | 30 mins |
|  | **Wait-list control group:** Research assistant contacts you to review your health & wellbeing | 15-minute phone call |
| **Week-20** | Questionnaires emailed to all participants to complete online* | 30-40 mins |
|  | **Treatment program group:** Complete audio-recorded feedback interview with research assistant | Up to 60 minutes |
|  | **Wait-list control group:** Research assistant contacts you to review your health & wellbeing | 15-minute phone call |
| **Week-32** | Questionnaires emailed to all participants to complete online* | 30-40 mins |
|  | **Treatment program group:** trial clinician contacts you for brief check in | 15 minutes |
|  | **Wait-list control group:** Offered access to 16-session treatment program^ | Approximately one session per week; 90 mins for the first session, 60 mins thereafter |

*If you prefer, paper copies of the study questionnaires can be posted to you. ^Plus additional questionnaires to complete 10-weeks and 20-weeks after starting the program

There are no additional costs associated with participating in this research project, nor will you be paid. All treatments and appointments involved in the study will be provided to you free of charge. You will be reimbursed for any reasonable travel, parking, meals, and other expenses associated with study participation (up to $50 per appointment).

If you are enrolled into the trial, and with your permission, we will send a letter to your local doctor letting them know that you have started the study.

***Neuroimaging (optional).***

If you live in, or can travel to, Melbourne, Victoria, we will also invite you to have Magnetic Resonance Imaging (MRI) scans of your brain at the Melbourne Brain Centre (University of Melbourne, Parkville) on two occasions. If you enrolled into the treatment group, we would invite you to have a scan before starting the program and after completing it. If you enrolled into the wait-list control group and decide to try the treatment program after Week 32, we would also invite you to have a scan before and after the program. The neuroimaging study will allow us to examine what effects DSCATT has on the brain and whether our treatment program influences these. This part of the study is optional, and you can still take part in the treatment program if you decide not to have the MRI scans.

You will be in the MRI scanner for approximately 45-60 minutes each time, during which we will collect images of your brain structure and function (changes in blood flow) as you lie still. During the scan, we will also measure your blood oxygen with a device placed on your finger, and your breathing via a belt worn around your abdomen. For part of the time, you will be asked to perform a task where you will see words on a screen and make judgments about them by pressing a button. The specific details of the task will be explained to you on the day of your scan, and we will train you on a computer beforehand to ensure you are familiar with the task requirements. After the scan, you will also be asked to complete a questionnaire related to the task. In total, you will need about 2 hours for the neuroimaging part of the study— including preparation time (e.g., task training, review of MRI safety screen, general set-up), time in scanner and time to complete the task-related questionnaire.

Prior to each MRI scan, a member of our research team and a radiographer from the Melbourne Brain Centre will do an MRI safety screen to ensure that it is suitable for you to have the scan. For example, we will ask you some questions to check that you do not have metal of any sort in your body, because of surgery or an injury. We may be required to access your relevant medical records and share them with the Melbourne Brain Centre Imaging Unit (University of Melbourne) for the purpose of this screen. Though some people may find lying in the MRI scanner uncomfortable, they do not pose any risks that we know of as long as you have been properly screened.

The MRI scans collected for this study are designed specifically for research purposes, and not for diagnostic or clinical purposes. However, scans with abnormalities will be flagged by the radiographer for review by a specialist. If the specialist identifies a significant abnormality that requires further clinical investigation, then the study’s medical monitors will be informed. The study doctor will discuss these findings with you and arrange a referral to appropriate clinical services. Your clinical care team will also be informed.

***Health economic evaluation (optional)***

With your permission, the researchers would also like to collect data regarding your health service use from the Medicare Benefits Schedule (MBS) and the Pharmaceutical Benefits Scheme (PBS) via Services Australia. This is so we can see how many medical appointments you attend, which medications you take, and how much these services cost you. This will provide an indication of how much it costs to have your illness treated outside of the trial, and also monitor treatment changes you receive whilst you are a participant in the trial. We will ask for your MBS & PBS data for the period 3-months prior to you being randomised into the trial, up until your Week-32 study questionnaires (about 11 months in total). The data that is provided will include the date of service, MBS/PBS item number, name of test or medication, and associated charges. It does not include the name of doctors or specialists you saw, or which clinic you attended. Please refer to the Services Australia Participant Information Document and Participant Consent Form for an example of information that will be provided for purposes of this study.

Services Australia is not involved in this research project other than to provide the information that you have consented to the release of, should you decide to participate in this study. Services Australia has confirmed that this research and any associated documents have received approval from a Human Research Ethics Committee (HREC) that is registered with and operates within guidelines set out by the National Health and Medical Research Council (NHMRC). You will be asked to sign a separate consent form authorising the study to access your Services Australia information, see the separate Services Australia Participant Information Document and Participant Consent Form.

**4 Other relevant information about the research project**

The study will be conducted at the Austin Hospital in Heidelberg, VIC, however, we will also offer appointments via Telehealth so people from regional areas and interstate can participate. The researchers are assessing if they can enrol 120 participants into this study.

**5 Do I have to take part in this research project?**

Participation in any research project is voluntary. If you do not wish to take part, you do not have to. If you decide to take part and later change your mind, you are free to withdraw from the project at any stage. If you do decide to take part, you will be given this Participant Information and Consent Form to sign and you will be given a copy to keep.

Your decision whether to take part or not to take part, or to take part and then withdraw, will not affect your routine treatment, your relationship with those treating you, or your relationship with Austin Health, The University of Melbourne, Monash University, or King’s College.

**6 What are the alternatives to participation?**

You do not have to take part in this research project to receive treatment. Your treating doctor and other specialists/health care providers can discuss what options are available with you.

**7 What are the possible benefits of taking part?**

We cannot guarantee you will receive any benefits from participating in this study as we are still exploring if this type of treatment is useful for people with DSCATT. If it does not benefit you directly, it may help us find out if this type of treatment is helpful for people who suffer from DSCATT, in what way does it help, and if it is worthwhile investigating it in a larger study.

**8 What are the possible risks and disadvantages of taking part?**

Based on previous research investigating these types of psychology treatments for other medical conditions, they have not been associated with serious side effects. However, there may be side effects that the researchers do not expect or do not know about which could be mild, moderate, severe, or serious. Tell study staff immediately about any new or unusual symptoms that you get. If a severe side effect or reaction does occur, one of the study doctors may need to stop your involvement in the trial. They will discuss the best way of managing any side effects with you.

It is possible that you may become upset, distressed, frustrated, or fatigued because of your participation in this study. If this does occur, the clinician providing your treatment will adjust the therapy to help you with any of these issues. You will also be provided a questionnaire to complete 20-weeks after starting the treatment program which asks you about side effects you believe the therapy may have caused.

This research project involves asking if you use illicit drugs. If you do, that information will be stored in a re-identifiable (or coded) format. If Austin Health is required to disclose that information by law, it may be used against you in legal proceedings or otherwise.

If you consent to participate in the optional **neuroimaging** component of the study, please note that the MRI scanner is limited for space and makes loud noises while it is in operation. It is therefore possible that you may experience some discomfort from having to lie still inside the MRI scanner and listening to the scanner noise. You will be provided with headphones to lessen the volume of the scanner noise. You may also experience a sense that your vision is rotating or a slight dizziness when first entering the MRI scanner, although this should be temporary. You will also have access to a communication buzzer which you can squeeze at any time to stop the scan.

**9 What if new information arises during this research project?**

Sometimes during the course of a research project, new information becomes available about the treatment that is being studied. If this happens, one of the study doctors will tell you about it and discuss with you whether you want to continue in the research project. If you decide to withdraw, the study doctor will make arrangements for your regular health care to continue. If you decide to continue in the research project, you may be asked to sign an updated consent form.

Also, on receiving new information, the study doctor might consider it to be in your best interests to withdraw you from the research project. If this happens, they will explain the reasons and arrange for your regular health care to continue.

**10 Can I have other treatments during this research project?**

One of the advantages of the type of treatment in this study is that it can be used alongside medications and other treatments you may already using for DSCATT or other medical conditions. However, if you use other psychology treatments, we ask that you stop these whilst you are completing the treatment program (the study doctors, in collaboration with you, and your treating team if needed, will determine if it is appropriate for you to do so). This is so we have a better chance of assessing the effects of the treatment program we are studying in isolation, as opposed to mixing it with other similar therapies.

Whilst you are participating in this research project, we encourage you to tell study staff about any treatments or medications you may be taking, including over-the-counter medications, vitamins or herbal remedies, acupuncture, or other alternative treatments. We also recommend you tell them about any changes to these during your participation in the treatment program.

**11 What if I withdraw from this research project?**

If you decide to withdraw from the project, please notify a member of the research team before you withdraw. This notice will allow that person or the study investigators to discuss any health risks or special requirements linked to withdrawing.

If you do withdraw your consent during the research project, study staff will not collect additional personal information from you, although personal information already collected will be retained to ensure that the results of the research study can be measured properly and to comply with law. You should be aware that data collected up to the time you withdraw will form part of the research project results. If you do not want them to do this, you can indicate your decision (yes/no) on the Withdrawal of Consent form you will be asked to sign when withdrawing from the study.

**12 Could this research project be stopped unexpectedly?**

This research project may be stopped unexpectedly for a variety of reasons. These may include reasons such as:

• Unacceptable side effects

• The treatment was shown not to be effective

• The treatment was shown to work and does not need further testing

• Not enough people express interest to participate

• Decisions made in the commercial interests of the sponsor or by local regulatory/health authorities.

**13 What happens when the research project ends?**

If you would like to continue with the treatment once the study is over, please discuss with your trial clinician. They will be able to provide guidance about how best to access this type of treatment going forward.

If you would like a summary of the results found in this study, please let a member of the research team know. We can send you a letter summarising the findings once the data analysis and any publications have been finalised.

**Part 2 How is the research project being conducted?**

**14 What will happen to information about me?**

The collection use and disclosure of your health information (including physical, mental, or psychological health) is governed by the Health Records Act 2001 (Vic) (HR Act) and Privacy and Data Protection Act 2014 (Vic) (PDP Act). By signing the consent form, you consent to the researchers, study doctors and relevant research staff collecting and using personal information about you for this research project. Your personal information will only be used for the purpose of this research project, and it will only be disclosed with your permission, except as required by law.

Information collected from you during the research study will be stored by the researchers in a ‘re -identifiable’ format. This means that identifying information will be removed from your participant file and replaced with a code so that only the researchers know how to identify you. Your study questionnaires will be sent to you electronically and stored securely by a software called REDCap (Research Electronic Data Capture). Only the researchers, study doctors and study staff approved to work on the project by the Austin Health Human Research Ethics Committee (HREC) will have access to your participant file. Any identifying information that is collected (for example, your contact details and those of your emergency contact) will be stored separately from your study questionnaires and other research data collected in this project.

Information about you may be obtained from your health records held at Austin Health for the purpose of this research. In addition, information about your participation in this research project will be recorded in Austin Health’s medical record system, for example, the dates that you attend appointments. By signing the consent form, you agree to the study team accessing your Austin Health medical records if they are relevant to your participation in this research project.

Information obtained from you during the research project are subject to inspection (for the purpose of verifying the procedures and the data) by the relevant authorities and authorised representatives of the Sponsor, Austin Health (the institution relevant to this Participant Information Sheet), The University of Melbourne, or as required by law. By signing the Consent Form, you authorise release of, or access to, this confidential information to the relevant study personnel and regulatory authorities as noted above.

In accordance with Victorian privacy and other relevant laws, you have the right to request access to your information collected and stored by the research team. You also have the right to request that any information with which you disagree be corrected. Please contact the study team member named at the end of this document if you would like to access your information.

The NHMRC strongly encourages **data sharing** between researchers who may also be investigating the same illness or other related aspects. This allows researchers to collaborate and ultimately increase knowledge about the illness in question – often in less time than if they were working independently. In this study, you have the option to provide *‘extended consent’* which means data collected from you (e.g., your online questionnaires, or neuroimaging data) may be shared with other research groups investigating DSCATT if requested. Their projects would also have to be approved by a registered Human Research Ethics Committee (HREC) before the data was shared. It is important to note that any data shared would be non-identifiable, meaning all of your identifying details would be removed.

All data collected in this study (including electronic and paper copies) will be stored securely for a minimum of 7 years after publication of the primary paper outlining the study findings. Thereafter, paper copies will be destroyed, however, electronic data files may be kept in perpetuity as advised by the Austin Health Office for Research and The University of Melbourne. This will facilitate data sharing with other researchers in future, as described in the paragraph above. Please note, these data files will not contain your identifying details (for example, your name or contact details). If you do not consent to data sharing, your data will be destroyed 7 years after publication of the primary paper.

It is anticipated that the results of this research project (including direct quotes from your treatment feedback interview) will be published and/or presented in a variety of forums. In any publication and/or presentation, information will be provided in such a way that you cannot be identified, except with your permission.

**15 Complaints and compensation**

If you suffer any injuries or complications as a result of this research project, you should contact a member from the research study as soon as possible, so you can be assisted with arranging appropriate medical treatment. If you are eligible for Medicare, you can receive any medical treatment required to treat the injury or complication, free of charge, as a public patient in any Australian public hospital. If you require compensation for any injuries incurred as a result of participation in this research study, you may be able to seek compensation through the court services.

**16 Who is organising and funding the research?**

This research project is being led by Professor Richard Kanaan (Principal Investigator; University of Melbourne) in collaboration with other researchers from The University of Melbourne, Austin Health, Monash University and King’s College in London*.*

The University of Melbourne has received government funding from the National Health and Medical Research Council (NHMRC) to conduct this research project. No member of the research team will receive a personal financial benefit from your involvement in this research project (other than their ordinary wages).

You will not benefit financially from your involvement in this research project even if, for example, information you provide proves to be of commercial value to the affiliated institutions.

In addition, if knowledge acquired through this research leads to discoveries that are of commercial value to the researchers or their institutions, there will be no financial benefit to you or your family from these discoveries.

**17 Who has reviewed the research project?**

All research in Australia involving humans is reviewed by an independent group of people called a Human Research Ethics Committee (HREC). The ethical aspects of this research project have been approved by the HREC of Austin Health (project reference number HREC/94244/Austin-2023).

This project will be carried out according to the *National Statement on Ethical Conduct in Human Research (2007)*. This statement has been developed to protect the interests of people who agree to participate in human research studies.

**18 Further information and who to contact**

The person you may need to contact will depend on the nature of your query.

If you want any further information concerning this project or if you have any medical problems which may be related to your involvement in the project (for example, any side effects), you can contact the principal study doctor on (03) 9496 3351 or any of the following people:

**Project coordinator/Research assistant**

| Name | [Name of project coordinator] |
| --- | --- |
| Position | Project coordinator |
| Telephone | (03) 8344 0189 |
| Email | dscatt-project@unimelb.edu.au |

**Clinical contact person/s**

| Name | [Name of trial clinician/s] |
| --- | --- |
| Position | Trial clinician |
| Telephone | (03) 9035 7126 |
| Email | dscatt@austin.org.au |

If you have any complaints about any aspect of the project, the way it is being conducted or any questions about being a research participant in general, then you may contact:

**Complaints contact person**

| Position | Complaints Officer |
| --- | --- |
| Telephone | (03) 9496 3566 |
| Email | feedback@austin.org.au |

**Reviewing HREC approving this research study**

| Reviewing HREC name | Austin Health Human Research Ethics Committee |
| --- | --- |
| HREC Executive Officer | Manager, Discovery & Innovation Unit |
| Telephone | (03) 9496 4090 |
| Email | [research@victri.org.au](mailto:research@victri.org.au) |

**Consent Form -** *Adult providing own consent*

| **Title** | Feasibility of an adjunctive intervention for Debilitating Symptom Complexes Attributed to Ticks (DSCATT) – a randomised, waitlist-controlled pilot and feasibility trial |
| --- | --- |
| **Short Title** | FIND trial |
| **Protocol Number (Version)** | 1 (3.1) |
| **Project Sponsor** | Austin Health |
| **Principal Investigator** | Professor Richard Kanaan |

**Consent Agreement**

► I have read the Participant Information Sheet or someone has read it to me in a language that I understand.

► I understand the purposes, procedures and risks of the research described in the project.

► I give consent for my therapy sessions and the post treatment feedback interview to be audio recorded.

► I give consent for parts of my post treatment feedback interview to be quoted in any publication or presentation arising from this research, once any identifying features have been edited out.

► I give consent for my medical records held by other healthcare providers to be sent to Austin Health for the purposes of my participation in this research study.

► I have had an opportunity to ask questions and I am satisfied with the answers I have received.

► I freely agree to participate in this research project as described and understand that I am free to withdraw at any time during the project without affecting my future health care.

🞎 YES

🞎 NO

► **OPTIONAL:** I give permission for non-identifiable data collected from me during this research project to be shared with other researchers who may be investigating DSCATT and/or other related conditions

► **OPTIONAL:** I consent to participating in the neuroimaging component of this study, and for my medical records (held by Austin Health or other healthcare providers) to be accessed and shared with the Melbourne Brain Centre Imaging Unit (University of Melbourne) for the purposes of the MRI safety screen.

🞎 YES

🞎 NO

► I understand that I will be given a signed copy of this document to keep.

**Declaration by Participant – for participants who have read the information**

| Name of Participant (please print) _________________________________________________  Signature _______________________________ Date _______________________________ |
| --- |

| Declaration - for participants unable to read the information and consent form  Witness to the informed consent process  Name (please print) __________________________________________________________  Signature _______________________________ Date ______________________________  * Witness is not to be the Investigator, a member of the study team or their delegate. Witness must be 18 years or older. |
| --- |

**Declaration by Study Doctor/Senior Researcher^†^**

I have given a verbal explanation of the research project, its procedures and risks and I believe that the participant has understood that explanation.

|  | | | | | | |
| --- | --- | --- | --- | --- | --- | --- |
|  | Name of Study Doctor/  Senior Researcher^†^ (please print) | |  | | |  |
|  | | | | | |  |
|  | Signature |  | | Date |  |  |
|  | | | | | | |

^†^ A senior member of the research team must provide the explanation of, and information concerning, the research project.

Note: All parties signing the consent section must date their own signature.

**Consent via telehealth (if applicable)**

- Consent was obtained using telehealth with *[Name of Participant]* whose photographic identification was sighted by the Investigator who observed the Participant’s signature being written
- Participant’s signed consent form received by the Investigator on [DD/MMM/YYYY].
- Consent was obtained using telehealth with *[Name of Investigator]* whose photographic identification was sighted by the Participant who observed the Investigator’s signature being written
- Discussed with *[Participant]* via telephone on [DD/MMM/YYYY] and received signed consent form on [DD/MMM/YYYY]. Signed by *[Investigator].*

|  | | | | | | |
| --- | --- | --- | --- | --- | --- | --- |
|  | Name of Participant (please print) | |  | | |  |
|  | | | | | | |
|  | Signature |  | | Date |  |  |
|  | | | | | | |

| For participants unable to read the information and consent form  Witness to the informed consent process  Name (please print) __________________________________________________________  Signature _______________________________ Date ______________________________  * Witness is not to be the Investigator, a member of the study team or their delegate. Witness must be 18 years or older. |
| --- |

|  | | | | | | |
| --- | --- | --- | --- | --- | --- | --- |
|  | Name of Study Doctor/  Senior Researcher^†^ (please print) | |  | | |  |
|  | | | | | |  |
|  | Signature |  | | Date |  |  |
|  | | | | | | |

† A senior member of the research team must provide the explanation of and information concerning the research project.

Note: All parties signing the consent section must date their own signature.

**Form for Withdrawal of Participation -** *Adult providing own consent*

| **Title** | Feasibility of an adjunctive intervention for Debilitating Symptom Complexes Attributed to Ticks (DSCATT) – a randomised, waitlist-controlled pilot and feasibility trial | | |
| --- | --- | --- | --- |
| **Short Title** | | FIND trial |  |
| **Protocol Number (Version)** | | 1 (3.1) |  |
| **Project Sponsor** | | Austin Health |  |
| **Principal Investigator** | | Professor Richard Kanaan |  |

**Declaration by Participant**

I wish to withdraw from participation in the above research project and understand that such withdrawal will not affect my routine treatment, my relationship with those treating me or my relationship with *The University of Melbourne, Austin Health, Monash University* or *King’s College*

I permit the researchers to use data that has already been collected from me and include it in the analyses and results of this project. 🞎 YES 🞎 NO

|  | | | | | | |
| --- | --- | --- | --- | --- | --- | --- |
|  | Name of Participant (please print) | |  |  |  |  |
|  | | | | | | |
|  | Signature |  | | Date |  |  |
|  | | | | | | |

*In the event that the participant’s decision to withdraw is communicated verbally, the Study Doctor/Senior Researcher will need to provide a description of the circumstances below.*

|  |
| --- |

**Declaration by Study Doctor/Senior Researcher^†^**

I have given a verbal explanation of the implications of withdrawal from the research project and I believe that the participant has understood that explanation.

|  | | | | | | |
| --- | --- | --- | --- | --- | --- | --- |
|  | Name of Study Doctor/  Senior Researcher^†^ (please print) | |  | | |  |
|  | | | | | |  |
|  | Signature |  | | Date |  |  |
|  | | | | | | |

^†^ A senior member of the research team must provide the explanation of and information concerning withdrawal from the research project.

Note: All parties signing the consent section must date their own signature.
