# Supplementary material for: Feasibility of an adjunctive INtervention for Debilitating symptom complexes attributed to ticks (FIND): study protocol for a randomised, waitlist-controlled feasibility trial
Source: BMJ Open. 2026 Mar 10;16(3):e112627. doi: 10.1136/bmjopen-2025-112627 (PMC12983826; doi:10.1136/bmjopen-2025-112627)
Supplement: online supplemental table 1 [file bmjopen-16-3-s002.docx]

#### **Supplemental material**

#### **Table S1.** Session outline of treatment intervention

| Session Number | Content | Corresponding chapters in patient manual |
| --- | --- | --- |
| Session 1 | - Establish therapeutic alliance - Consent and expectations - Assessment - Initial case formulation and overall goals for therapy (introduce “choice point”) | - Ch. 1 Background *(provided as reading homework)* - Ch. 2 The Experience of Symptoms *(provided as reading homework)* |
| Session 2 | - Explore treatment rationale: - Education on the neurobiological underpinning of the program and how the brain is involved in symptom experience. - Understanding the role of stress and emotions in symptom experience - Exploring targets for treatment      - Introduce Acceptance and Commitment Therapy (ACT) model - Discuss how a psychological therapy can help manage physical symptoms - Understand core principles of ACT and ACT as collaborative process - Getting back to what's important despite the uncertainty of illness. - Use “choice point’ to illustrate key elements of ACT (e.g., workability, values)      - Revisit initial formulation incorporating ACT concepts (e.g., choice point - workability, triggers/maintaining factors) | - Ch. 2 The Experience of Symptoms (*provide for homework if not completed last session)* - Ch. 3 Model of Therapy *(provided as reading homework)* |
| Session 3 | - Review understanding of ACT model - Introduce daily routine monitoring - Discuss the benefits of increasing activity - Explore paced activity versus Boom and Bust - Introduce sleep monitoring (optional) | - Ch. 4 Daily routine *(provided as reading homework)* - Ch. 5 Increasing levels of activity *(provided as reading homework)* - Ch. 11 Improving sleep - optional *(provided as reading homework)* |
| Session 4 | - Review daily routine monitoring - Optional: Review sleep monitoring - Establish initial (simple) activity program based on values - Introduce values and workability | - Ch. 5 Increasing levels of activity *(provided as reading homework)* - Ch. 6 Values *(provided as reading homework)* |
| Session 5 | - Review progress of initial activity schedule (homework) - Identify and discuss barriers to progress - Establish another initial (simple) activity program based on values - Optional: Review sleep monitoring - Introduce ‘unhooking’: - How our minds get hooked by difficult thoughts/feelings/sensations, leading to unworkable behaviour - Use simple metaphors | - Ch. 5 Increasing levels of activity - Ch. 6 Values - Ch. 8 Unhooking *(provided as reading homework)* |
| Session 6 | - Introduce goal setting (guided by values) - Agree on a larger ‘committed action plan’ for initial goals - Expand on ‘unhooking’ - Do experiential exercises - teach defusion skills - Practice in-session - Discuss how this can be applied to everyday life/values/goals | - Ch. 6 Values - Ch. 7 Goals *(provided as reading homework)* - Ch. 8 Unhooking - Ch. 9 Living with Uncertainty *(provided as reading homework)* |
| Session 7 | - Review committed action plan and revise if necessary. - Identify barriers that impede progress - Introduce concepts of struggling with uncertainty versus willingness - Review experiential unhooking techniques, and introduce additional strategies if needed | - Ch, 7 Goals - Ch. 8 Unhooking - Ch. 9 Living with uncertainty *(provided as reading homework)* |
| Session 8 | - Review committed action plan and revise if necessary. - Review understanding of willingness and introduce experiential exercises - Introduce self-compassion and how it can be used to regulate stress/distress | - Ch. 7 Goals - Ch. 9 Living with uncertainty - Ch. 10 Self- compassion *(provided as reading homework)* |
| Session 9 - 14 | **Modules to repeat:**   - Discuss patients ongoing/new concerns and apply ACT strategies - Review principles of ACT model and brain’s role in experience of symptoms as needed - Review targets for therapy, particularly behavioural goals - Reinforce values and assess workability of thoughts/behaviours - Set goals, troubleshoot barriers, and agree on a committed action plan each week - Practice ‘unhooking’ from difficult thoughts/feelings/sensations - Evaluate futile control strategies that show up, and explore willingness as an alternative | **Optional modules to introduce (if appropriate):**   - Ch. 11 Improving sleep (if not yet covered in earlier sessions) - Ch. 12 Managing thinking & memory difficulties - Ch. 13 Managing relationships |
| Session 15-16 | - Preparing for discharge - Review case formulation - Review treatment rationale and summarise key modules - Review ways to maintain progress - Goals post-intervention - Managing setbacks | - Ch. 14 Wrapping up |
