# Supplementary material for: Feasibility of an adjunctive INtervention for Debilitating symptom complexes attributed to ticks (FIND): study protocol for a randomised, waitlist-controlled feasibility trial
Source: BMJ Open. 2026 Mar 10;16(3):e112627. doi: 10.1136/bmjopen-2025-112627 (PMC12983826; doi:10.1136/bmjopen-2025-112627)
Supplement: online supplemental file 2 [file bmjopen-16-3-s003.docx]

## **Supplemental material**

## **Semi-structured interview guide for feedback interviews**

**Introduction**

• Confirm identity and introduce interviewer

• Confirm willingness to participate in interview

• Remind of audio recording and confidentiality of interview and analysis

• Remind that interview is being voice recorded for transcription and analysis

• Explanation of objective of determining the acceptability, value/benefits and/or other effects of the treatment program

•Remind participant that there are no right/wrong answers – we are interested to hear their honest perspectives told in their own words

• Outline structure and duration of the interview (free discussion ~30-60 mins, with some prompts)

• **Any questions before we begin?**

**Opening question:**

*• “I’d like to hear your experiences with the treatment program for DSCATT you have just you taken part in…looking back on the sessions that you had, can you tell me a bit about what you thought of the treatment? Where would you like to start?”*

**General probes (when indicated):**

• Encourage the participant to talk freely about the treatment without suggesting topics. Listen for reference to components of the study and when appropriate, prompt to explore them one by one by saying: **“You mentioned ‘X’…what was that like? Can you tell me more about it? Why do you think that is?”**

If participant seems keen to talk about their current health or experiences and it doesn’t seem relevant to understanding their perspectives on the treatment, acknowledge what they are saying and then try to orient them back to talking about their views of the treatment itself, if necessary say, **“I can see that there’s a lot to talk about, but because we only have a little while together, I’d like us to go back to talking about your experience of the treatment program.”**

**Targeted probes (when appropriate):**

**•** I’d like to hear what you **liked** about the treatment program, and also what you **didn’t like**.

- If participant provides examples for the above, explore **why** they liked/didn’t like ‘X’

• It sounds like you’re saying that ‘X’ was significant, can you tell me more about ‘X’?

•You mentioned that ‘X’ was ‘good/bad’…why do you think that is the case?

•Was there anything else about the experience that you can tell me about?

**If not covered already:**

•What effect has the program had on you, if any?

- In what way? Why do you think that is the case?

•Did you notice any changes in your health or wellbeing whilst doing the program, or not?

- Why do you think that might be?

•Did you notice changes in any other areas of your life?

- Can you give me any examples?

• When the participant is not generating any more information in response to the above probes or time is nearly up: **“is there anything else you’d like to add regarding your experiences with, or views about the treatment program?”**

To conclude, thank the participant for their time and important contribution to the study. Remind them how the interview data will be treated and used (audio file of the interview transcribed word for word – although names will be removed; analysed alongside other participant interview transcripts to look for commonalities and differences between the group).

**Any final questions?** Remind participant to reach out to a member of the study team if they have any other questions or queries.
