# Supplementary material for: Feasibility of an adjunctive INtervention for Debilitating symptom complexes attributed to ticks (FIND): study protocol for a randomised, waitlist-controlled feasibility trial
Source: BMJ Open. 2026 Mar 10;16(3):e112627. doi: 10.1136/bmjopen-2025-112627 (PMC12983826; doi:10.1136/bmjopen-2025-112627)
Supplement: Supplementary file 4 [file bmjopen-2025-112627.reviewer_comments.pdf]

PEER REVIEW HISTORY

BMJ Open publishes all reviews undertaken for accepted manuscripts. Reviewers are asked to complete a checklist review form and are provided with free text boxes to elaborate on their assessment. These free text comments are reproduced below.

ARTICLE DETAILS

Title (Provisional)

Study protocol for Feasibility of an adjunctive INtervention for Debilitating symptom complexes attributed to ticks (FIND) - a randomised, waitlist controlled feasibility trial

Authors

Kanaan, Richard; Macdonell, Richard; Long, Michelle; Richardson, Robert J; Rogers, Caitlyn; Mcmanus, Mualla; Braat, Sabine; Zaloumis, Sophie; Mihalopoulos, Cathrine; Chatterton, Mary Lou; Gibney, Katherine B; Oliver, Gina; Wilson, Sarah J; Chalder, Trudie

---

VERSION 1 - REVIEW

**Reviewer**	**1**
**Name**	**Halperin, John**
**Affiliation**	**Overlook Medical Center, Neurosciences**
**Date**	**05-Dec-2025**
**COI**	**None**

The authors are to be commended for designing a pilot study to assess a reasonable therapeutic intervention (Acceptance and Commitment Therapy, which includes elements of CBT) in patients with a common symptom complex that fits the rubric of Medically Unexplained Symptoms, while avoiding much of the controversial baggage associated with inappropriate attribution of the symptoms to a specific pathophysiology. While patients with these symptoms are often resistant to the notion that treatment focusing on psychologic aspects might be beneficial, the deliberate inclusion of patient advocacy groups in the protocol design may at least partially help overcome this challenge. The study is well designed and acknowledges many of the obvious potential pitfalls. My suggestions primarily relate to clarification.

1. The question of acceptability to control patients of deferring the intervention for 8 months when they've already been symptomatic for at least 6 is not explicitly addressed and may limit recruitment. Was this addressed in discussions with patient groups?

2. The issue of including patients already using other psychological therapies is potentially problematic. Inclusion of such individuals - who already accept a psychological approach -

might result in a population bias. Additionally, pausing such treatment in control subjects during the study period might suggest an apparent treatment benefit if the control subjects' symptoms worsen due to treatment absence. If such patients are included, results should probably be stratified into those with and without such prior interventions, and outcomes assessed separately.

3. Subjects are required to have laboratory evidence of prior infection with a tick-borne pathogen. Would it be helpful to include a group with tick bite, these symptoms but none of these positive serologies?

4. Primary outcome: the primary outcome actually includes 3 – practicality, acceptability and demand. While this would be problematic in a definitive trial, it can be forgiven in an exploratory feasibility study.

5. The neuro-imaging is obviously very exploratory in nature. That said, its rationale would be strengthened by inclusion of some relevant background data explaining why fMRI during the Stroop test might be informative

6. A small point - Table 3 (3.g.ii) uses the word 'neuropathy' to explain "weakness, nerve pain, numbness and tingling". Most such patients lack evidence of neuropathy and it would probably be best to omit this term

---

Reviewer 2

Name Raffetin , Alice

Affiliation Intermunicipal Hospital Centre Villeneuve Saint Georges, Infectious Diseases and Tropical Medicine

Date 23-Dec-2025

COI None

This protocol is an absolutely necessary study to improve the care of patients experiencing medical wandering in Australia, as well as in other countries, whether endemic or not for Lyme borreliosis, where many patients report suffering from DSCATT, with no evidence to date in the scientific literature of effective treatments. Acceptance and Commitment Therapy (ACT) has been proven effective in randomized trials for the management of functional syndromes such as chronic pain and fibromyalgia. The hypothesis that this type of ACT-based intervention could improve patients' quality of life therefore deserves serious consideration, and the authors propose to test it through this well-designed randomized feasibility trial with sound methodological quality. Nevertheless, please find below few comments regarding both form and content in order to improve the trial and anticipate potential sources of bias:

Introduction:

- P.5, Line 2: Illness or Disease? "most famous tick-borne disease", isn't it? But DSCATT is an illness, isn't it?

- P.5 Line 3: Sensu lato is not in italics, just "Borrelia burgdorferi"

- P.5 Line 45-46: It should also be specified that the existence of this clinical entity as a distinct disease remains uncertain, as it falls within the spectrum of medically unexplained symptoms (MUS). However, in the absence of a clear medical explanation, this uncertainty should benefit patients: even without established pathophysiological mechanisms, it remains appropriate to offer symptomatic and supportive care aimed at improving quality of life.

Methods:

- P.8:

o Will KMF be involved in the development of the study tools?

o Do they adhere to the study messages and to the protocol? Do they endorse the ACT framework?

o Are they involved in the drafting of the study protocol? Ok, answer p17 !

o Will you verify that patients recruited through this advocacy group genuinely adhere to the protocol? This point is important, as some previous studies have been biased by the inclusion of « false participants » deliberately adressed to undermine trials, notably in an international study on persistent symptoms following a COVID-19 infection. => I think you will do it if I understand well p 11 (screening), do I ?

o Will patients included in the study be able to interact with patient representatives during the course of the protocol? It can be very constructive if patient representatives are constructive.

o Will a patient satisfaction questionnaire be implemented? If so, at which study endpoints will it be administered?Is it the one in Table 1 (acceptability?)

- P.9:

o Will there be differences between Telehealth or face-to-face sessions? Have you planned any subgroup analyses to account for potential differences in delivery modes?

o What is the expected time commitement for completing the homework assignements? Will non-compliance constitute an exclusion criterion?

- P. 10: Conventional psychological therapies are part of standard care. It may therefore be challenging to ask participants to discontinue them. Would it be preferable to exclude patients who have already undergone psychological therapies for the same indication, as this could influence the outcomes? Patients who engage in psychological therapies may differ systematically from those who have never received such interventions. Alternatively, have you considered planning a subgroup analysis to address this potential source of bias?

- The use of mixed methods is an excellent approach and will enhance the overall quality and interpretability of the study

- P.14: Could you please specify the level of precision that is considered acceptable or expected for the study outcomes?

Conclusion: How do you plan to inform study participants and the general public about the results of your study? Scientists are often criticized for insufficient communication, which is regrettable as it frequently contributes to misinformation and misinterpretation of scientific findings.

Table 2: You may wish to consider adding a generic quality-of-life instrument such as the EQ-5D-5L or the SF-12 to facilitate comparisons with previous studies. Did the patient representatives involved in the steering committee agree to the administration of this number of questionnaires?

Table 3 : serological testing for Babesia and Anaplasma are not validated tools… Q-fever is maybe not transmitted by ticks, there is a controversy…You should test for borrelia and TBEV only (and less expensive…)

Semi-structered interviews :

- You could consider adding a question addressing the patients' social environment: how did relatives or close contacts perceive the patient's participation in the study and its impact on their health outcomes?

- If you were a physician, what would you propose to your patients in routine clinical practice? Would you recommend ACT as a therapeutic option?

References: Maybe you could add recent meta-analysis about ACT?

VERSION 1 - AUTHOR RESPONSE

Reviewer: 1

1. The question of acceptability to control patients of deferring the intervention for 8 months when they've already been symptomatic for at least 6 is not explicitly addressed and may limit recruitment. Was this addressed in discussions with patient groups?

There is no perfect psychotherapy control condition, with waitlist tending to overestimate the benefits of the intervention, but also to present a disincentive to participation, which would tend to be conservative for a feasibility study such as this. The rationale for using a waitlist control group in this study is specified on page 2 of the PICF (included as Supplemental Material in this resubmission) and will be highlighted to participants during informed consent discussions. Participants will also be encouraged to continue with their treatment as usual during the waitlist period.

In addition, reasons why participants decline enrolment into the trial will be explored by the trial coordinator during the pre-screening process, where possible (see Table 1, Demand row, Qualitative column). We anticipate this will capture dissatisfaction with the waitlist component, where apparent, and thereby assist in gauging the feasibility of the trial design.

When developing the protocol, the length of the waitlist was discussed by the trial steering committee, including our patient representative. We agreed on a 32-week waitlist period to match the randomised therapy participants timeframe in the study (20-weeks to complete the intervention + 12-week follow-up). This will provide control data after therapy participants complete the intervention and explore if any benefits are retained versus treatment as usual.

2. The issue of including patients already using other psychological therapies is potentially problematic. Inclusion of such individuals - who already accept a psychological approach - might result in a population bias. Additionally, pausing such treatment in control subjects during the study period might suggest an apparent treatment benefit if the control subjects' symptoms worsen due to treatment absence. If such patients are included, results should probably be stratified into those with and without such prior interventions, and outcomes assessed separately.

To clarify, participants are only required to pause pre-existing psychological therapies when they are completing this study's 16-session treatment intervention. Participants randomised into the waitlist control arm may continue (or commence) psychological therapies, whilst they are waitlisted. This is outlined in the *'Standard care and addition to standard care'* section of the manuscript. We have however added an additional sentence in this section for further clarity: *"They* [participants] *may use other psychological therapies after they complete the intervention, if desired, or if they are randomised into the waitlist control arm."* Healthcare use throughout the trial, including psychological therapies, will be captured by a study specific resource use questionnaire. Heterogeneity of the results by prior intervention can be explored descriptively via subgroup analyses. This subgroup has been added to the manuscript (see 'Clinical outcomes' on page 16).

3. Subjects are required to have laboratory evidence of prior infection with a tick-borne pathogen. Would it be helpful to include a group with tick bite, these symptoms but none of these positive serologies?

This is an excellent suggestion, and if we obtain such a group we can do a subgroup analysis. However, as outlined in Table 3, Eligibility Criteria, participants are required to have evidence of a tick bite by EITHER:

a) Observation of a tick bite <u>OR</u>
b) serological results from a NATA-accredited laboratory consistent with a previous infection transmitted by ticks

We have therefore not routinely collected serology from those meeting criterion A. While many will provide this to us at their screening anyway, it is unlikely they will do so if all of their results are negative. If we have this evidence, we can do the analysis suggested.

4. Primary outcome: the primary outcome actually includes 3 – practicality, acceptability and demand. While this would be problematic in a definitive trial, it can be forgiven in an exploratory feasibility study.

Thank you for pointing this out. When designing this protocol, we elected to follow feasibility markers as outlined by Bowen et al., 2009; Lancaster, 2015; O'Cathain et al., 2015. We opted to use 'retention rate' as a primary outcome – see *'Feasibility outcomes'* section on page 14 – to inform the target sample size.

5. The neuroimaging is obviously very exploratory in nature. That said, its rationale would be strengthened by inclusion of some relevant background data explaining why fMRI during the Stroop test might be informative

Thank you for the suggestion. We have now added two paragraphs to the protocol, one on the rationale for the Stroop, and one for how we think it will respond to the intervention.

6. A small point - Table 3 (3.g.ii) uses the word 'neuropathy' to explain "weakness, nerve pain, numbness and tingling". Most such patients lack evidence of neuropathy and it would probably be best to omit this term

Many thanks for raising this important point. While we agree, the precise language was determined by our Delphi process, which included a number of non-clinicians. Amending the case criteria would require re-opening the modified Delphi we completed and asking Delphi participants to vote on changing this terminology, which is not realistic at this stage.

Reviewer: 2

Introduction:

- P.5, Line 2: Illness or Disease? "most famous tick-borne disease", isn't it? But DSCATT is an illness, isn't it?

Correct, DSCATT is considered an illness as per the Australian Government Department of Health who conceptualized the term. As such, when referring to Lyme disease in the opening paragraph of the introduction, we have amended 'tick-borne **illness'**, to 'tick-borne **disease'**

- P.5 Line 3: Sensu lato is not in italics, just "Borrelia burgdorferi"

We have amended this.

- P.5 Line 45-46: It should also be specified that the existence of this clinical entity as a distinct disease remains uncertain, as it falls within the spectrum of medically unexplained symptoms (MUS). However, in the absence of a clear medical explanation, this uncertainty should benefit patients: even without established pathophysiological mechanisms, it remains appropriate to offer symptomatic and supportive care aimed at improving quality of life.

Thank you for the suggestion. We have incorporated this into the introduction.

Methods:

o Will KMF be involved in the development of the study tools?
o Do they adhere to the study messages and to the protocol? Do they endorse the ACT framework?
o Are they involved in the drafting of the study protocol? Ok, answer p17 !

The study tools were developed in coordination with the trial consumer representative. KMF have assisted with recruitment, advertising the study through their channels.

o **Will you verify that patients recruited through this advocacy group genuinely adhere to the protocol? This point is important, as some previous studies have been biased by the inclusion of « false participants » deliberately addressed to undermine trials, notably in an international study on persistent symptoms following a COVID-19 infection. => I think you will do it if I understand well p 11 (screening), do I ?**

This is an intriguing point. Our trial therapists have raised concerns that some participants may have had other motivations for their involvement, though these were not necessarily those recruited from KMF. We have endeavoured to treat all participants equally, however, regardless of these concerns. Ours is a feasibility study, and we believe that the complex engagement of a community that may have doubts about the study is a critical part of the feasibility of a trial like this, rather than a distraction. All participants enrolled into the trial, whether recruited from patient advocacy groups or elsewhere, are assessed for engagement with the intervention via the number of sessions attended, reasons for

withdrawing from the intervention prematurely where apparent, and feedback provided in the post-treatment exit interview.

o Will patients included in the study be able to interact with patient representatives during the course of the protocol? It can be very constructive if patient representatives are constructive.

Participants are welcome to liaise with patient advocacy groups or the like whilst they are participating in the trial, however, this will not be formally monitored by the study's framework.

o **Will a patient satisfaction questionnaire be implemented? If so, at which study endpoints will it be administered? Is it the one in Table 1 (acceptability?)**

Yes, we have included a study specific, self-reported participant satisfaction questionnaire which will be administered at Week-20 (conclusion of the intervention). This is referenced in Table 1. Acceptability, quantitative column. We have also included a copy of the questionnaire as Supplemental Material as part of this resubmission.

o Will there be differences between Telehealth or face-to-face sessions? Have you planned any subgroup analyses to account for potential differences in delivery modes?

While the content delivered in face-to-face and Telehealth sessions will be the same, we acknowledge that the mode of delivery may influence the outcome. This subgroup has been added to the manuscript (see 'Clinical outcomes' on page 16). We anticipate that most participants will complete the intervention via Telehealth, however.

o What is the expected time commitment for completing the homework assignments? Will non-compliance constitute an exclusion criterion?

Time commitments for homework tasks are specified on page 3 of the PICF (included as Supplemental Material as part of this resubmission), and we have also added this to the manuscript – see *Therapy Arm* sub-section, page 10. Participants will not be withdrawn from the trial if they are not compliant with their homework tasks. This will however be explored in session by the trial therapist and homework completion recorded, so that it may be included in the analysis.

- P. 10: Conventional psychological therapies are part of standard care. It may therefore be challenging to ask participants to discontinue them. Would it be preferable to exclude patients who have already undergone psychological therapies for the same indication, as this could influence the outcomes? Patients who engage in psychological therapies may differ systematically from those who have never received such interventions. Alternatively, have you considered planning a subgroup analysis to address this potential source of bias?

This is an excellent point. Treatment histories will be collected during the screening visit by the trial physician, including if the participant has utilised psychological therapies. This could be reported in the primary results paper when describing the characteristics of the recruited sample. We have added this subgroup to the manuscript (see 'Clinical outcomes' on page 16).

- The use of mixed methods is an excellent approach and will enhance the overall quality and interpretability of the study

Thank you for this feedback – we're glad the reviewer is supportive of the study design we have selected.

- P.14: Could you please specify the level of precision that is considered acceptable or expected for the study outcomes?

The sample size is informed by the precision around the primary feasibility outcome of retention. In the sample size estimation paragraph (page 14), we state that "*Assuming 120 participants are randomised, if study completion (i.e., retention) is observed for 60% of these participants (72/120), then the two-sided 95% confidence interval (CI) of the true underlying retention rate is [51%, 69%]. The exact (Clopper-Pearson) method was used to obtain the 95% CI.*" This corresponds to a precision of ± 9%.

Conclusion: How do you plan to inform study participants and the general public about the results of your study? Scientists are often criticized for insufficient communication, which is regrettable as it frequently contributes to misinformation and misinterpretation of scientific findings.

We agree it is important to share the study results with participants and make accessible to the general public. Our study results dissemination plans are outlined in the *'Dissemination policy'* sub-section of the *'Ethics & dissemination section'* on page 18. Specifically, *"A lay, HREC-approved summary of the primary study results will be communicated to participants directly, if desired. A copy of this summary will also be uploaded onto the study's clinical trial registry listing (ANZCTR) and provided to the Australian Government Department of Health for their Tick bite diseases and symptoms attributed to tick bites website."* We further note that all outcomes from the study will be published 'open access', and though we appreciate these scientific publications are not aimed at the general public, we know that previous DSCATT project outcomes have been keenly examined by consumer organisations, and open access requested.

Table 2: You may wish to consider adding a generic quality-of-life instrument such as the EQ-5D-5L or the SF-12 to facilitate comparisons with previous studies. Did the patient representatives involved in the steering committee agree to the administration of this number of questionnaires?

The number of questionnaires were approved by all members of the steering committee, including our patient representative.

We elected to use the Assessment of Quality of Life-8D (AQoL-8D) given it is a validated, multi-attribute utility measure commonly used in Australia and has an Australian scoring algorithm. Furthermore, it was developed using very sound psychometric techniques to be more sensitive to psychosocial related domains of quality of life compared to the other instruments, such as EQ-5D-5L and the SF-12. These domains are of particular importance within this study context.

There are cross walk algorithms whereby AQoL-8D scores can be converted to EQ-5D-5L or SF-6D scores that we could consider using as a comparison in a sensitivity analysis. This approach would prevent further burden on participants by not adding more questionnaires.

Table 3 : serological testing for Babesia and Anaplasma are not validated tools... Q-fever is maybe not transmitted by ticks, there is a controversy...You should test for borrelia and TBEV only (and less expensive...)

For clarity, the DSCATT case definition (developed by a Modified Delphi process) which serves as part of our inclusion criteria, requires evidence of a tick bite by EITHER:

a) Observation of a tick bite <u>OR</u>
b) serological results from a NATA-accredited laboratory consistent with a previous infection transmitted by ticks

We do not routinely test trial candidates for any of the conditions listed in Table 3. Rather, positive tests for these conditions (undertaken in their previous medical work-up) indicate likely historical tick bites (exposure to ticks). We acknowledge the reviewer's concerns regarding Babesia, Anaplasma and Q-fever

testing to identify tick-transmitted diseases, however, testing in reference laboratories is offered in Australia and supported by healthcare guidelines (albeit the low prevalence of these TBDs is also noted). Further, this terminology was agreed upon during our Delphi process, and therefore, it is not realistic to amend at this stage.

Semi-structured interviews :
- You could consider adding a question addressing the patients' social environment: how did relatives or close contacts perceive the patient's participation in the study and its impact on their health outcomes?
- If you were a physician, what would you propose to your patients in routine clinical practice? Would you recommend ACT as a therapeutic option?

Thank you for these suggestions, however if we made these changes at this stage in the study they would impact only a minority of participants, probably too few to be of value.

References: Maybe you could add recent meta-analysis about ACT?

Thank you for the suggestion. We have provided two new references in the Introduction (see reference 19 & 20): Levin 2024 & Gloster 2020 – the latter a review of meta-analyses re. ACT

VERSION 2 - REVIEW

**Reviewer**	**1**
**Name**	**Halperin, John**
**Affiliation**	**Overlook Medical Center, Neurosciences**
**Date**	**01-Feb-2026**
**COI**	

I thank the authors for their responses. Since it appears the recruitment period has ended, I look forward to. reading the results

---

**Reviewer**	**2**
**Name**	**Raffetin , Alice**
**Affiliation**	**Intermunicipal Hospital Centre Villeneuve Saint Georges, Infectious Diseases and Tropical Medicine**
**Date**	**03-Feb-2026**
**COI**	

Thank you for the changes. The manuscript is clear enough to my point of view and of good scientific quality.
